# Association between thyroid cancer and cardiovascular disease: A meta-analysis

**DOI:** 10.3389/fcvm.2023.1075844

**Published:** 2023-03-03

**Authors:** Wen-Hsuan Tsai, Yi-Hong Zeng, Chun-Chuan Lee, Ming-Nan Chien, Sung-Chen Liu, Kuo-Liong Chien, Shih-Ping Cheng, Po-Jung Tseng, Ming-Chieh Tsai

**Affiliations:** ^1^ Division of Endocrinology and Metabolism, Department of Internal Medicine, Mackay Memorial Hospital, Taipei, Taiwan; ^2^ Department of Medicine, MacKay Medical College, New Taipei City, Taiwan; ^3^ Institute of Epidemiology and Preventive Medicine, National Taiwan University, Taipei, Taiwan; ^4^ Department of General Surgery, Mackay Memorial Hospital, Taipei, Taiwan; ^5^ Division of Cardiovascular Surgery, Department of Surgery, Hsin Chu Armed Force Hospital, Hsinchu, Taiwan

**Keywords:** thyroid cancer, cardiovascular disease, thyroxine, radioactive iodine, tyrosine kinase inhibitors

## Abstract

**Objective:**

To determine the association between thyroid cancer and coronary artery disease, atrial fibrillation, cerebrovascular disease, and cardiovascular disease mortality.

**Methods:**

The PubMed, Embase, and Cochrane Library databases were searched for eligible studies from inception to September 22, 2022. Keywords included “thyroid cancer”, “atrial fibrillation”, “coronary artery disease”, “cerebrovascular disease”, and “mortality”. Primary outcomes included the incidence of coronary artery disease, cerebrovascular disease, atrial fibrillation, and cardiovascular disease mortality among patients with thyroid cancer. Secondary outcomes included cardiovascular disease events among those with thyroid cancer that received or did not receive radioactive iodine or lenvatinib. Estimates were pooled using fixed- and random-effects meta-analysis.

**Results:**

A total of 771,220 patients who underwent thyroidectomy in 15 studies were included. Risk for cerebrovascular disease (risk ratio [RR] 1.15 [95% confidence interval (CI) 1.10–1.21]) and atrial fibrillation [RR 1.59 (95% CI: 1.45–1.73)] were significantly increased. Risk for coronary artery disease was significantly increased [RR 1.12 (95% CI: 1.08–1.17)] in the common effect model. Cardiovascular disease mortality associated with thyroid cancer was not significant [RR 0.93 (95% CI: 0.59–1.45)]. Radioactive iodine had a neutral effect on cardiovascular disease [RR 1.00 (95% CI: 0.87–1.16)], and there was no beneficial nor harmful effect among different RAI doses.

**Conclusions:**

Thyroid cancer was significantly associated with a higher risk for cerebrovascular disease and atrial fibrillation; however, the hazard risk was not different between patients with and without radioactive iodine treatment. Thyroid cancer treatment should be individualized considering the potential harms and benefits to cardiovascular health.

## Introduction

The incidence of thyroid cancer (TC) is ranked 11th according to the GLOBOCAN 2018 estimates of cancer incidence and mortality produced by the International Agency for Research on Cancer (1). The incidence of TC has been increasing over the past few decades (2) and, if recent trends persist, TC may become the fourth most common cancer by 2030 in the United States (3). Such an increase is likely due to improved detection and diagnosis, which largely or totally reflects the over-diagnosis of indolent disease (2). The survival rate, particularly for papillary carcinoma, is extremely high (>98% five-year survival rate in Europe or North America), resulting in comparatively low mortality rates (2). The 10-year relative survival rates for patients with papillary, follicular, and Hurthle cell carcinomas were 93%, 85%, and 76%, respectively (4). Primary management of TC includes surgery, radioactive iodine (RAI), and thyroid-stimulating hormone (TSH) suppression therapy, according to American Thyroid Association (ATA) risk stratification system (5). In 2022, a randomized controlled trial of 776 patients with low-risk TC demonstrated that after thyroidectomy, active surveillance without RAI was non-inferior to RAI regarding the occurrence of functional, structural, and biologic events at third year (6). Active surveillance has been advocated as an alternative to active management for low-risk papillary thyroid micro-carcinoma (PTMC) (7). Despite good prognosis, 4%–16% of patients with PTMC develop recurrent disease, with many of which developing distant metastasis (8–10). A cohort of 407 patients with PTMC revealed that patients with lymph node metastasis who did not receive I-131 had a 5-year recurrence-free survival of 42.9% vs. 93.2% (*P* < 0.0001) for patients who received I-131 (11). On the other hand, patients with radioiodine refractory (RR)-DTC have a 5-year survival rate of as low as 10% (12). Tyrosine kinase inhibitors, such as sorafenib and lenvatinib, were approved for the treatment of patients with RR-DTC (13, 14). To sum up, the treatment strategy for TC should be individualized.

173,710 patients with TC from the Surveillance, Epidemiology, and End Results (SEER) database showed that 29.1% and 21.7% of death were attributable to TC and cardiovascular disease (CVD) (15). Older age, male sex, non-white race, unmarried status, and advanced stage were independent predictors of CVD mortality, while receiving surgery and radiotherapy were protective against CVD mortality (15). On the other hand, 30,778 patients with TC from Taiwan's National Health Insurance Research Database revealed that the primary cause of death was TC mortality (31.2%), followed by other malignancy-related mortality (29.9%) and CVD mortality (12.3%) (16). Some research has suggested that the management of TC could lead to long-term cardiovascular risks. For example, studies have demonstrated that patients with TC had higher odds (hazard ratio [HR] 1.16 [95% confidence interval (CI) 1.05–1.28]) for CVD (17) and higher odds [HR 1.29 (CI: 1.06–1.57)] for atrial fibrillation (Af) (18) compared with the general healthy population. In contrast, however, another study failed to find an association between a high risk for CVD or Af and patients with TC (19). The association between tyrosine kinase inhibitors (TKIs) and CVD in patients with TC is still lacking.

Owing to the increasing incidence and relatively good prognosis of TC, an updated and comprehensive quantitative review is needed to assess the long-term effects on the cardiovascular system among patients with TC after thyroidectomy, RAI and TKIs. We aimed to examine the incidence of coronary artery disease (CAD), Af, cerebrovascular disease and CVD mortality in patients with TC; and the risk for CVD among patients who underwent RAI or lenvatinib (TKI).

## Methods

### Information sources and search strategy

Two authors (W.H.T and M.C.T) independently identified articles published before September 22nd, 2022, that investigated and reported on “thyroid cancer”, “atrial fibrillation”, “coronary artery disease”, “cerebrovascular disease”, and “mortality”. (Supplementary Table S1) by systematically searching the PubMed (Medline), Embase, and Cochrane Library databases. The authors screened the titles and abstracts of studies and identified those for inclusion eligibility using approaches prescribed by the Preferred Reporting Items for Systematic Reviews and Meta-analyses and Meta-analysis of Observational Studies in Epidemiology reporting guidelines (20).

### Eligibility criteria

Two reviewers initially screened the titles and abstracts of all retrieved articles for eligibility. Studies with the following characteristics were excluded: target population(s) not related to TC; data that did not address the outcome of interest; reviews, meta-analyses, or commentaries. The inclusion criteria included all patients with TC that received either thyroidectomy, thyroxine, RAI or TKIs. After initially screening articles for inclusion based on title and abstract, the full text of each was reviewed. Disagreements were resolved by the third author. The PECOS framework was demonstrated in Supplementary Table S2 (21).

### Data extraction and quality assessment

The following information was extracted from each study: year of publication; author(s); country; number of participants (TC/control); proportion of women; age at diagnosis; mean years' follow-up; statistical analysis; and adjusted variable and outcomes with hazard ratio (HR). The Risk of Bias in Non-randomized Studies‒Exposure (ROBINS-E) was used to assess the risk of bias (22).

### Data synthesis and statistical analysis

Outcome ascertainment was variably defined as CAD, cerebrovascular disease, Af, and CVD mortality. In the first analysis, pooled risk estimates of CAD, cerebrovascular disease, Af and CVD mortality between patients with TC and the general healthy population were calculated. The risk for CVD between TC patients with and without RAI or lenvatinib was also examined. We extracted the number of CVD adverse events among the clinical trials and calculated the difference in the observed percentages of patients. We used logistic regression to perform the odds ratio with 95% confidence interval (CIs) of the CVD adverse events among clinical trials. When ≥ 2 studies assessed the same outcome, results were pooled using both fixed- and random-effect meta-analysis, which specifically accounted for heterogeneity among studies. Extracted pooled HRs for individual outcomes were combined to construct summary pooled HRs. Heterogeneity was assessed using standard chi-squared tests and the *I*^2^ statistic, for which *I*^2^ > 75% indicated substantial heterogeneity. Random-effect meta-regression was performed to investigate sources of heterogeneity (mean age, women proportion, follow up years).

Linear dose responses were modeled using the method by Greenland and Longnecker with RRs (95% CIs) from RAI dose categories to determine the association with the risk of CVD (23). Two studies (24, 25) were included for information regarding the association. Doses were defined as the mean exposure in each reported category. The width of the adjacent interval was used as an upper or lower cut-off value with open-ended extreme categories. The Wald test for departure from linearity association was conducted and significance was defined at *P* < 0.10 (2-sided) (26) which leads to further non-linear analysis. The pairwise analyses were significant based on *P* < 0.05. Non-linear dose-response relationship was assessed using restricted cubic splines with three knots at 10%, 50%, and 90% percentiles of the distribution.

Bias secondary to small study effects was assessed using visual inspection of funnel plots but could not be examined by Egger test plots because there was fewer than 10 studies. Statistical significance was defined as two-sided *P* < 0.05. All analyses were performed using R version 3.5.1 (R Project for Statistical Computing).

## Results

### Study identification and selection

**Figure 1:**
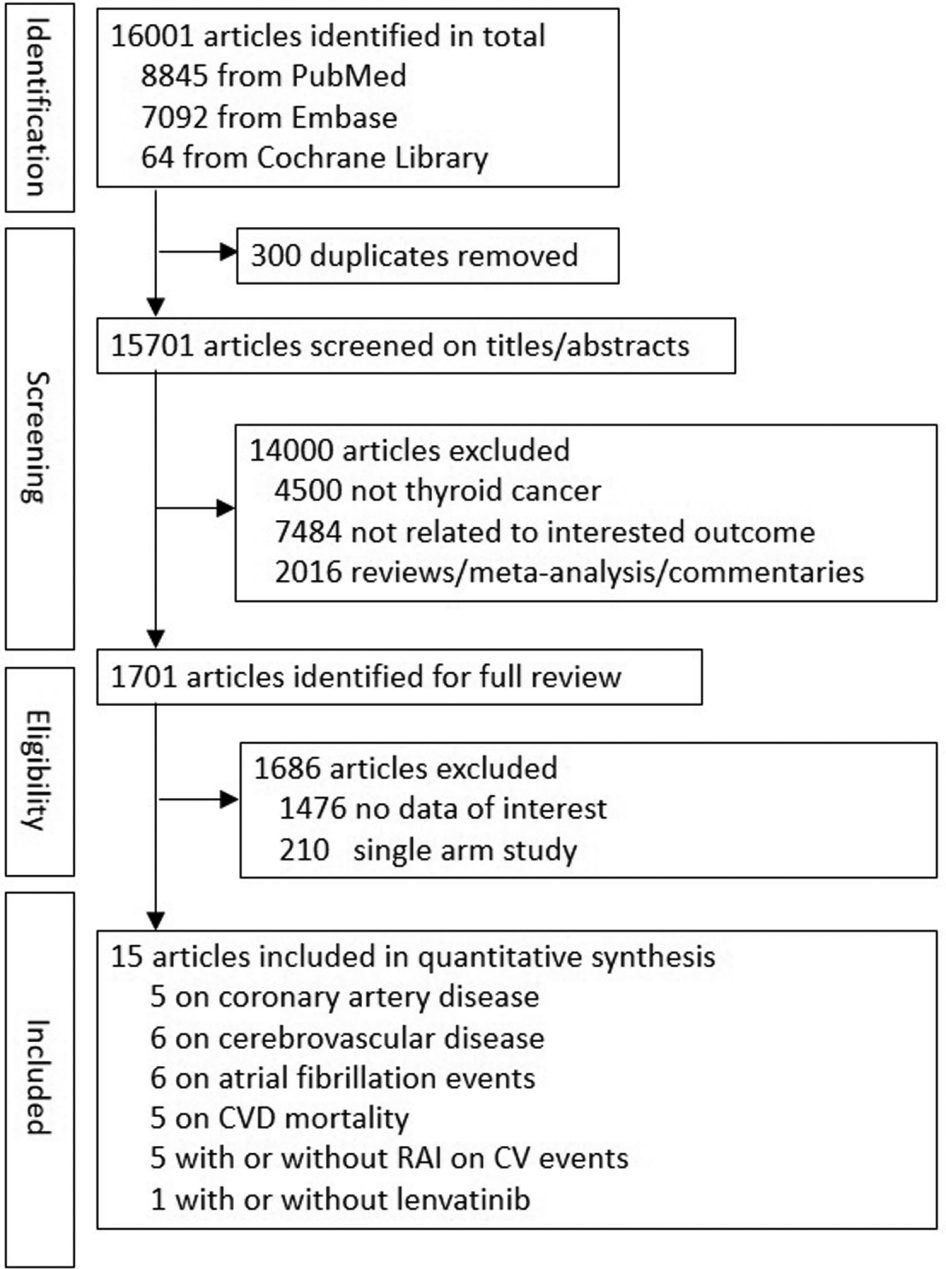
The process of literature search based on the PRISMA statement.

As shown in Figure 1, 16,001 studies were retrieved in the literature search, of which 300 articles were duplicates, leaving 15,701 articles to assess for inclusion. An additional 14,000 studies were excluded based on title and/or abstract, and another 1,686 articles excluded based on full-text review, which resulted in 15 studies for quantitative analysis. Among these 15 studies, 3 of the articles included patients without thyroidectomy (17, 24, 27), with the percentage of 22%, 35.5%, and 2.2%, respectively. There were five studies (17, 19, 28–30) including 420,175 patients for evaluation of CAD; six studies (17, 19, 27–30) including 439,468 patients for evaluation of cerebrovascular disease; six studies (17, 19, 27–29, 31) including 385,150 patients for evaluation of Af; five studies (15–18, 25) including 310,475 patients for evaluation of CVD mortality; five studies (6, 24, 25, 27, 32) including 48,328 patients to examine the risk for CVD between patients with TC who did and did not undergo RAI therapy. One randomized clinical trial with 392 patients was included to examine the risk for CVD between patients with TC who did and did not receive lenvatinib (13). Five studies (17, 18, 28, 31, 33) were included to assess the association between levothyroxine dose or TSH level and CVD, Af and CVD mortality. The country, participant number, women proportion, mean age, follow-up years, adjusted variables, and outcome ascertainment for the included studies are summarized in Table 1. Risk of bias for all 15 individual studies are listed in Supplementary Table S3.

**Table 1:** Characteristics of studies included in meta-analysis stratified by the risk of CAD, cerebrovascular disease, Af, CVD mortality, different TSH level (mU/liter) control, levothyroxine dose (μg/day) response, RAI (with or without) and cumulative RAI dose, lenvatinib (with or without).

Study	Country	Number (DTC/Control)	Women%, Age (SD), Follow up (yrs)	Preexisting CVD	Adjusted variable	Outcome HR
The risk of CAD among patients with DTC compared with healthy control						
Pajamäki, 2018	Finland	901/4,485	81, 48 (16), 18.8	The results did not change when the subjects with a prevalent CVD were excluded	Prevalent CVD	0.94 (0.78–1.12)
Suh, 2019	Korea	182,419/182,419	84.2, 47 (11.3), 4.3	Excluded	Age, sex, income, monthly insurance, residence, disability	1.15 (1.1–1.22)
Toulis, 2019	UK	3,009/11,303	75.8, 50.7 (16.1), 4.8	Excluded	Age, BMI, sex, smoking status, Townsend deprivation index, hypertension, diabetes and lipid-lowering medication	1.04 (0.8–1.36)
Zoltek, 2020	Sweden	6,000/	73.7, 54	NA	NA	SIR*: 0.97 (0.84–1.11)
Izkhakov, 2019	Israel	5,677/23,962	78.6, 50 (16), 7.6	3%/8.2%	Age, sex, hypertension, diabetes mellitus, current smoking, dyslipidemia and previous cardiovascular disease	1.2 (1.08–1.34)
The risk of cerebrovascular disease among patients with DTC compared with healthy control						
Pajamäki, 2018	Finland	901/4,485	81, 48 (16), 18.8	The results did not change when the subjects with a prevalent CVD were excluded	Prevalent CVD	0.98 (0.78–1.24)
Blackburn, 2017	USA	3,706/15,587	77.9, 46	Excluded	Age, sex, birth state, BMI, race, CCI	<40 y/o: 2.26 (0.75–6.78) >40 y/o: 1.20 (0.88–1.64)
Suh, 2019	Korea	182,419/182,419	84.2, 47 (11.3), 4.3	Excluded	Age, sex, income, monthly insurance, residence, disability	1.15 (1.09–1.22)
Toulis, 2019	UK	3,009/11,303	75.8, 50.7 (16.1), 4.8	Excluded	Age, BMI, sex, smoking status, Townsend deprivation index, hypertension, diabetes and lipid-lowering medication	1.34 (1.05-1.72)
Izkhakov1, 2019	Israel	5,677/23,962	78.6, 50 (16), 7.6	3%/8.2%	Age, sex, hypertension, diabetes mellitus, current smoking, dyslipidemia and previous cardiovascular disease	1.19 (1.04–1.37)
Zoltek, 2020	Sweden	6,000/	73.7, 54	NA	NA	SIR*: 1.14 (1.01–1.29)
The risk of atrial fibrillation among patients with DTC compared with healthy control						
Hesselink, 2015	Netherlands	518/1,563	74.4, 48.6 (14), 10.5	1.2%/1.6%	Age, sex, HTN, BMI, Hx of CAD and HF	2.47 (1.55–3.95)
Blackburn, 2017	USA	3,706/15,587	77.9, 46	Excluded	Age, sex, birth state, BMI, race, CCI	1.05 (0.66–1.66)
Pajamäki, 2018	Finland	901/4,485	81, 48 (16), 18.8	The results did not change when the subjects with a prevalent CVD were excluded	Prevalent CVD	1.29 (1.06–1.57)
Suh, 2019	Korea	182,419/182,419	84.2, 47 (11.3), 4.3	Excluded	Age, sex, income, monthly insurance, residence, disability	Levothyroxine <115 μg/day, HR: 1.43 (1.27–1.62); Levothyroxine 115–144 μg/day, HR: 1.41 (1.26–1.58); Levothyroxine 145–169 μg/day, HR: 1.66 (1.49–1.85); Levothyroxine ≥170 μg/day, HR: 1.78 (1.60–1.98)
Toulis, 2019	UK	3,009/11,303	75.8, 50.7 (16.1), 4.8	Excluded	Age, BMI, sex, smoking status, Townsend deprivation index, hypertension, diabetes and lipid-lowering medication	1.71 (1.36-2.15)
Zoltek, 2020	Sweden	6,000/	73.7, 54	NA	NA	SIR*: 1.66 (1.41–1.94)
The risk of CVD mortality among patients with DTC compared with healthy control						
Hesselink, 2013	Netherlands	524/1,572	74.4, 48.6 (14), 10.5	2.5%/2.9%	Age, sex, HTN, BMI, Hx of CAD and HF	3.35 (1.66–6.74)
Pajamäki, 2018	Finland	901/4,485	81, 48 (16), 18.8	The results did not change when the subjects with a prevalent CVD were excluded	Prevalent CVD	0.73 (0.58–0.92)
Kim, 2020	Korea	4,082/12,246	78.6, 46, 3	3.2%/3.2%	BMI, socioeconomical, smoking, alcohol, HTN, DM and dyslipidemia	0.5 (0.22–1.16)
Lu, 2021	Taiwan	30,778 /92,334	75.8, 50.7 (16.1), 5.67	2.2%/4.5%	Age, sex, ischemic heart disease, ischemic stroke, hemorrhagic stroke, hyperlipidemia, DM, HTN, and occupation	0.56 (0.42–0.76)
Du, 2021	USA	163,553/	76.7, 48, 8.5	NA	NA	PTC: 0.57 (0.55–0.59) FTC: 1.14 (1.06–1.23) Hürthle cell TC: 1.47 (1.30–1.67)
Adjusted HRs for CVD by with or without RAI						
Pajamäki, 2018	Finland	901/4,485	81, 48 (16), 18.8	The results did not change when the subjects with a prevalent CVD were excluded	Prevalent CVD	CVD: TC with I131 vs. control: HR 1.18 (1.05–1.31); TC without I131 vs. control: HR 1.07 (0.85–1.34)
Blackburn, 2017	USA	3,706/15,587	77.9, 46	Excluded	Age, sex, birth state, BMI, race, CCI	1.13 (0.99–1.29)
Lin, 2017	Taiwan	5,052/5,052 (with/without RAI)	84.6, 49.4 (13.8)	11.7%/11.8%	Age, sex, hypertension, hyperlipidemia, diabetes, COPD, CAD, alcohol-related illness, asthma	0.97 (0.73–73.30)
Suh, 2019	Korea	182,419/182,419	84.2, 47 (11.3), 4.3	Excluded	Age, sex, income, monthly insurance premiu, residence, disability	CVD: TC with I131 vs. control: HR 1.18 (1.11–1.26); TC without I131 vs. control: HR 1.15 (1.06–1.25)
Suh, 2019	Korea	182,419/182,419	84.2, 47 (11.3), 4.3	Excluded	Age, sex, income, monthly insurance premiu, residence, disability	Ischemic stroke: TC with I131 vs. control: HR 1.13 (1.06–1.21); TC without I131 vs. control: HR 1.18 (1.09–1.28)
Kim, 2020	Korea	2,533/2,312 (with/without RAI)	78.6, 46, 3	7.1%/7.6%	Age, sex, body mass index, socioeconomical, smoking, alcohol, levothyroxine, and history of HTN, DM, dyslipidemia, CVD	CVD: HR 0.87 (0.71–1.07); ischemic stroke: HR 0.83 (0.51–1.34); ischemic heart disease: HR 0.90 (0.71–1.13)
Leboulleux, 2022	France	389/387 (with/without RAI)	82.8, 52.4, 3	NA	Randomized controlled trial	OR: 0.52 (0.05–5.61) with RAI: 1 heart attack, without RAI: 1 transient ischemic stroke, 1 stroke
Kao, 2021	Taiwan	11,889/1,421	80, 47.3, 5.16	Excluded	Age, sex, DM, CKD, hyperlipidemia, and modified Charlson comorbidity index score	0.99 (0.84–1.16)
Adjusted HRs for CVD by with or without tyrosine kinase inhibitors						
Schlumberger, 2015 (SELECT): Lenvatinib	USA, Europe, Asia, and Australia	261/131	49, 63, 1.5	NA	Randomized controlled trial	3.54 (1.03–12.13)
Brose, 2014 (DECISION): Sorafenib	18 countries	207/210	52.3, 63, 0.9	NA	Randomized controlled trial	Sorafenib: 2 CAD, 0 Af Placebo: 0 CAD, 2 Af
Adjusted HRs for CVD by TSH (mU/liter) compared with healthy control						
Pajamäki, 2018	Finland	901/4,485	81, 48 (16), 18.8	The results did not change when the subjects with a prevalent CVD were excluded	Prevalent CVD	TSH < 0.1, HR: 1.27 (1.03–1.58); TSH: 0.1–0.5, HR: 1.04 (CI 0.83–1.31); TSH > 0.5, HR: 1.31, (0.98–1.77)
Adjusted HRs for CVD by Levothyroxine dose (μg/day)						
Suh, 2019	Korea	182,419/182,419	84.2, 47 (11.3), 4.3	Excluded	age, sex, income, monthly insurance premiu, residence, disability	CAD: Levothyroxine <115 μg/day, HR: 1.41 (1.26–1.58); Levothyroxine 115–144 μg/day, HR: 1; Levothyroxine 145–169 μg/day, HR: 1.61 (1.45–1.8); Levothyroxine ≥170 μg/day, HR: 2.15 (1.94–2.39)
Suh, 2019	Korea	182,419/182,419	84.2, 47 (11.3), 4.3	Excluded	age, sex, income, monthly insurance premium, residence, disability	Ischemic stroke: Levothyroxine <115 μg/day, HR: 1.31 (1.16–1.47); Levothyroxine 115–144 μg/day, HR: 1; Levothyroxine 145–169 μg/day, HR: 1.63 (1.46–1.82); Levothyroxine ≥170 μg/day, HR: 2.15 (1.94–2.39)
Adjusted HRs for atrial fibrillation by TSH (mU/liter)						
Wang, 2015	USA	465/306 (TSH ≤ 0.4/TSH > 0.4)	73.8, 48 (14), 6.5	Af excluded	age, sex, RAI, and ATA risk category	TSH ≤ 0.4 v.s. TSH > 0.4, HR 1.02 (0.54–1.91)
Adjusted HRs for atrial fibrillation by each 10-fold decrease in TSH level						
Hesselink, 2015	Netherlands	518/1,563	74.4, 48.6 (14), 10.5	1.2%/1.6%	age, sex, HTN, BMI, Hx of CAD and HF	1.01 (0.71–1.44)
Adjusted HRs for CVD mortality and total mortality by each 10-fold decrease in TSH level						
Hesselink, 2013	Netherlands	524/1,572	74.4, 48.6 (14), 10.5	2.5%/2.9%	age, sex, HTN, BMI, Hx of CAD and HF	CVD mortality: HR 3.08 (1.32–7.21); Total mortality: 1.43 (0.97–2.12)

* SIR: standardized incidence ratio.

### Risk for CAD, cerebrovascular disease, AF, and CVD mortality compared between patients with TC and healthy controls in the general population

**Figure 2:**
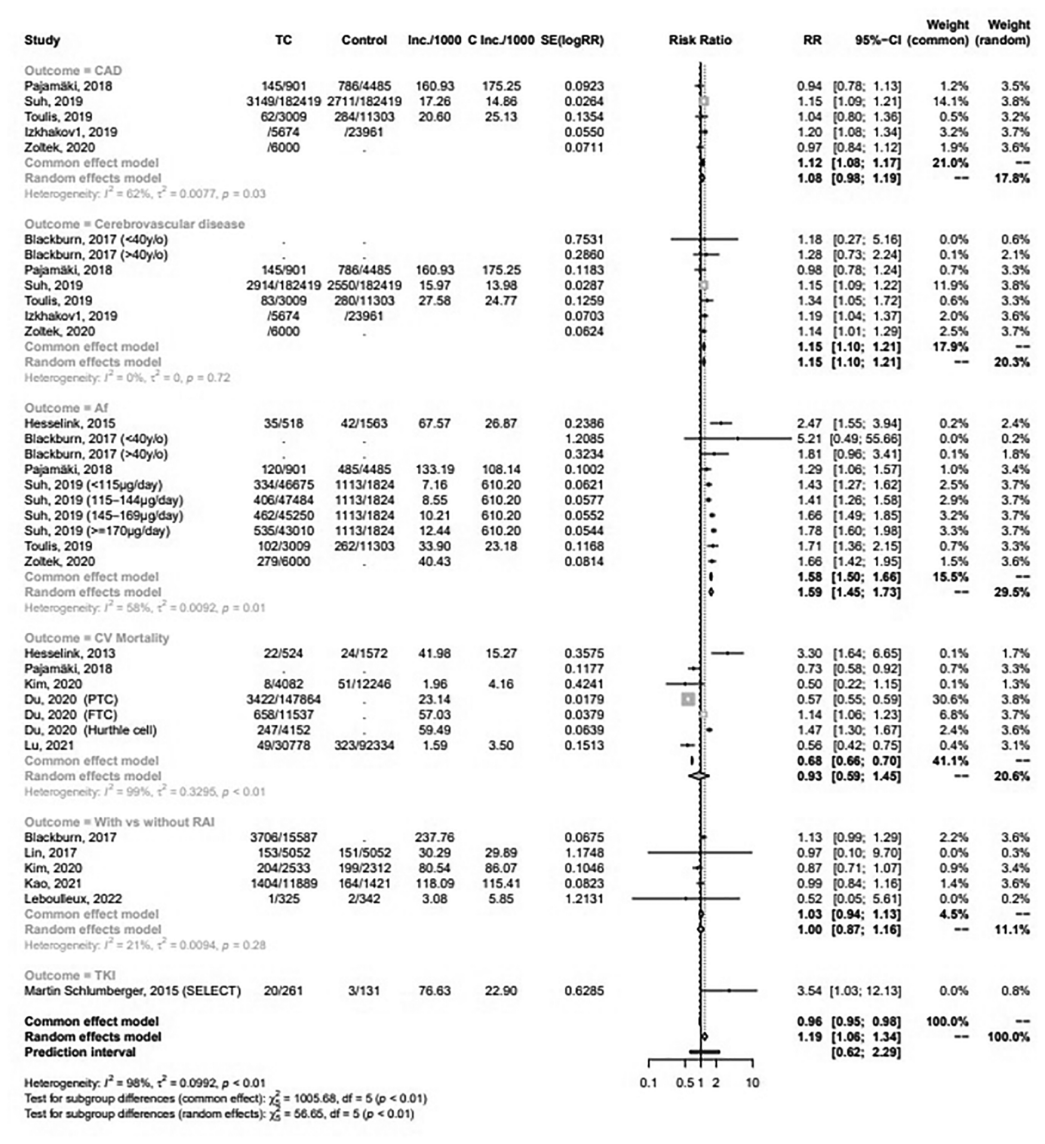
Forest plots for meta-analysis of the association between thyroid cancer and CAD; cerebrovascular disease; Af; CVD mortality; and the risk of CVD among patients with TC with and without RAI or lenvatinib.

Meta-analytic pooling of hazard estimates of CAD between patients with TC and the general healthy population in 5 studies (Figure 2) revealed a non-significantly increased risk [RR 1.08 (95% CI 0.98‒1.19)] in the random effect model. However, a significantly increased risk [RR 1.12 (95% CI: 1.08‒1.17)] was noted in the common effect model. Patients with TC had significantly higher risk of cerebrovascular disease [RR 1.15 (95% CI: 1.10‒1.21)] and Af [RR 1.59 (95% CI: 1.45‒1.73)] (Figure 2). Of the 5 studies (Figure 2) that investigated CVD mortality associated with TC, there was no significantly decreased risk [RR 0.93 (95% CI: 0.59‒1.45)].

### Risk for CVD among DTC patients who did and did not undergo RAI or lenvatinib

**Figure 3:**
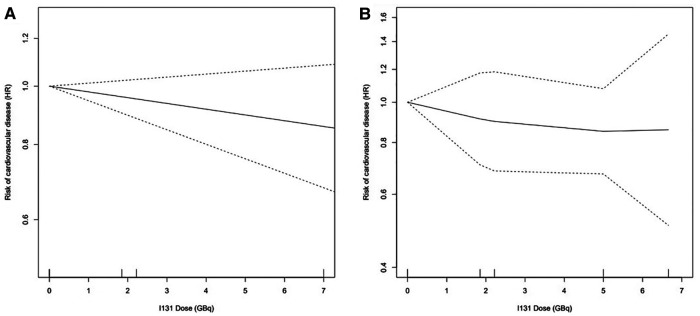
Dose-response association between RAI dose and the risk of CVD: (**A**) linearity risk ratio (RR) per 1 GBq, 0.98 [95% CI: 0.95–1.01], *P *= 0.20; (**B**) non-linearity RR per 1 GBq, 0.94 [95% CI: 0.78–1.32], *P *= 0.52.

The pooled risk for CVD between patients with TC that received or did not receive RAI in 5 studies (Figure 2) demonstrated no significantly increased risk [RR 1.00 (95% CI: 0.87‒1.16)]. A randomized controlled trial (RCT) (Figure 2) suggested a significantly increased risk of lenvatinib for CVD [RR 3.54 (95% CI: 1.03‒12.13)]. Figure 3A showed the dose-response association of RAI cumulative dose and the incidence of CVD. The linear model was demonstrated by the solid line and 95% CI was represented by the dotted lines. Data from 2 cohorts (24, 25) with a dose range of 0 to 6.66 GBq demonstrated an adverse linear dose-response association between RAI and risk of CVD [RR per 1 GBq, 0.98 (95% CI: 0.95–1.01)] with no evidence for departure from linearity (Wald test, *P *= 0.2). Figure 3B was non-linear associated model [RR per 1 GBq, 0.94 (95% CI: 0.78–1.32), *P *= 0.52]. The findings demonstrated no beneficial nor harmful effect on CVD among different RAI doses.

### Association between levothyroxine dose or TSH level and the risk for CVD or CVD mortality

Systematic review of the association between CVD risk and different dosages of levothyroxine is summarized in Table 1. Both the risk for CAD and ischemic stroke were increased in TC patients treated with gradually higher dosages of levothyroxine after thyroidectomy. In addition, each 10-fold decrease in geometric mean TSH level could significantly predict CVD mortality, with an HR of 3.08 (95% CI: 1.32‒7.21) after adjusted, but not all-cause mortality.

### Publication bias, study heterogeneity, and meta-regression

The risk of publication bias was found to be non-significant for studies addressing CAD, cerebrovascular disease, Af, and CVD mortality (Supplementary Figure S1). Meta-regression analysis demonstrated no statistically significant association between risk for CAD, cerebrovascular disease, Af and CVD mortality and mean age, women proportion, and follow up years (Table 2).

**Table 2:** Meta-regression for the association between baseline characteristics, including mean age, the proportion of women, and follow up years, and the risk of (A) CAD; (B) cerebrovascular disease; (C) Af; (D) CVD mortality among patients with TC compared with general health population.

	Slope	95% CI		*τ*^2^ (%)	*I*^2^ (%)	*R*^2^ (%)	*p* value
**Coronary artery diseases**							
Age	−0.01	−0.08	0.05	0.93	66.28	0	0.57
The proportion of women	0.01	−0.03	0.05	0.84	60.93	0	0.44
Follow up years	−0.01	−0.04	0.02	0.13	25.27	73.45	0.23
**Cerebrovascular diseases**							
Age	−0.02	−0.05	0.01	0.11	15.39	82.18	0.15
The proportion of women	0.005	−0.04	0.05	0.86	57.73	0	0.71
Follow up years	−0.01	−0.05	0.03	0.1	15.98	0	0.33
**Atrial fibrillation**							
Age	−0.01	−0.06	0.03	1.23	64.01	0	0.58
The proportion of women	−0.003	−0.03	0.03	12.15	68.64	0	0.83
Follow up years	−0.01	−0.04	0.02	1.12	69.64	6.32	0.45
**CVD mortality**							
Age	−0.06	−0.36	0.24	38.52	99.25	0	0.63
The proportion of women	−0.2	−0.47	0.06	23.06	98.67	30	0.11
Follow up years	0.02	−0.13	0.18	41.36	99.27	0	0.72

## Discussion

The present study showed that TC patients were prone to experience cerebrovascular disease and Af. The incidence of CAD and CVD mortality among TC patients did not appear to be different from the general healthy population. In addition, there was no significant association between RAI and CVD in patients with TC. However, according to limited evidence, lenvatinib may increase CVD risk.

3,822 patients from Utah Population Database (UPDB) demonstrated that male, overweight or obese, older at cancer diagnosis, TSH suppression therapy, distant metastases at cancer diagnosis, and a higher Charlson comorbidity index score were associated with an increased CVD risk among TC survivors (34). In our study, majority of patients were women and the mean age was between 40 and 50 years old. Both Izkhakov et al. (35) and Suh et al. (28) demonstrated a significantly increased CAD risk in patients with TC compared with the general healthy population. Pajamaki et al. (17) and Toulis et al. (29) showed no significant risk of CAD, but the population number of these two studies were smaller than the former two studies. Our study demonstrated a neutral risk of CAD in the random effect model, which was inconsistent with previous meta-analysis (36). However, in the common effect model, our study demonstrated a significantly higher risk of CAD. The difference was that we also included Zoltek's study (19), despite this study using standardized incidence ratios as the statistical strategy. Toulis et al. (29), Izkhakov et al. (35), and Suh et al. (28) reported a significantly higher risk of cerebrovascular disease, while Blackburn et al. (27) and Pajamaki et al. (17) showed no significant risk of cerebrovascular disease. The former three studies had higher population number than the latter two studies. Our study showed a significantly higher risk for cerebrovascular disease, which was consistent with previous meta-analysis (36). However, the hazard ratio that previous meta-analysis extracted from Toulis's study (29) was unadjusted. In addition, we included Blackburn's study (27). Among all the studies above, only Izkhakov et al. (35) included patients with preexisting CVD, while the others excluded patients with preexisting CVD. Besides, the proportion of CVD in placebo group was much higher than TC group in Izkhakov's study (8.2% vs. 3%) (35). Regarding Af, Hesselink et al. (31), Pajamäki et al. (17), Suh et al. (28) and Toulis et al. (29) indicated that the risk tended to increase among patients with TC; however, Blackburn et al. (27) could not find the difference. Only Hesselink's study (31) included patients with preexisting CVD, with higher proportion of CVD in placebo group than TC group (1.6% vs. 1.2%). Our result was consistent with another meta-analysis (37), which also indicated a significantly higher risk of Af. However, we excluded Abonowara's (38) study since this was a cross-sectional study rather than a cohort study.

The pathophysiological mechanism of increased cerebrovascular disease in patients with TC remains unclear. Suh et. al found a low incidence of Af in patients who developed ischemic stroke, which may suggest that Af is not a primary factor associated with ischemic stroke (28). Both alternatives of thyroid function, such as subclinical hyperthyroidism and hypothyroidism, could be possible reasons explaining the increased risk for CVD in patients with TC. For example, research had shown that subclinical hyperthyroidism increased left ventricular size (39, 40), systolic hypertension, diastolic dysfunction and reduce arterial elasticity (41). In addition, the pro-thrombotic effects related to subclinical hyperthyroidism have been reported to be involved in the development of hypercoagulability (38, 42–45). In contrast, hypothyroidism has also been linked to oxidation of low-density lipoprotein, induction of atherosclerosis, and consequent diastolic hypertension (46, 47). Finally, dysrhythmia (48–50), angina (48–50) and coronary heart disease (46, 47) during TC treatment may possibly contribute to the increased odds for CVD. Regarding TSH-suppression therapy and CVD incidence, a U-shaped relationship between either TSH concentration or cumulative dose of levothyroxine and CVD risk was observed (17, 28). Higher odds for CVD were noted in the end range of TSH-suppression therapy. Hesselink et al. (18) described the detrimental effect of 10-fold TSH level decreasing on CVD mortality, while there was still lack of evidence supporting the effect of TSH level on Af (31, 33). The discrepancy in results between these cohorts could be explained by different statistical methods, end points, and data acquisition methods.

Further investigations of CVD mortality rate demonstrated no difference after follow up of 4,082 patients with TC for 3 years (51). Pajamäki et al. (17) (901 patients follow up for 18.8 years) and Lu et al. (16) (30,778 patients follow up for 5.67 years) showed significantly lower risk of CVD mortality. In contrast to these studies, Hesselink et al. (18) studied 524 patients with 10.5 follow-up years and noted that CVD mortality risk increased after adjusting for cardiovascular risk factors. Higher proportion of CVD at baseline in placebo group than TC group was noted in Hesselink (18) and Lu's (16) studies. Whether the difference was owing to different CVD surveillance strategy was unknown. Our study suggested neutral risk of CVD mortality in patients with TC. Previous research suggested that RAI may trigger increased short-term intima media thickness in the arteries (52) or echocardiographic variability (53). A previous epidemiological cohort study investigating hyperthyroidism demonstrated that, after undergoing RAI treatment, the subsequent incidence of CVD may increase (54–57). However, other studies indicated that patients with TC who underwent RAI treatment had non-significant odds (range, 0.71‒1.29) for developing CVD compared with those without RAI (27, 51). Our meta-analysis revealed that TC patients receiving RAI treatment was not associated with a higher risk for CVD. The association between RAI dose and CVD incidence is controversial. One study suggested that the cumulative dose of RAI was associated with a gradually increased risk for Af (31). However, other studies failed to find an association between higher risk for stroke and RAI dose (24, 25). Our study revealed that there was no beneficial nor harmful effect among different RAI doses. The pathogenic mechanism between RAI treatment and CVD is also inconclusive. The major explanation of the discrepancies of CVD incidence with RAI therapy between patients with hyperthyroidism and TC may be that the underlying hyperthyroidism-related cardiotoxic effects may not be fully reversed even after restoring euthyroidism (55). On the other hand, a study showed that iodine-131 reduced cell proliferation and induced apoptosis of human cardiac muscle cells through the p53/Bax/caspase-3 and PIDD/caspase-2/t-BID/cytochrome c/caspase-3 signaling pathway (58). Since there is still lack of evidence, whether higher risk of CVD mortality in Hesselink's study (18) is due to higher RAI dose (200 mCi) than other studies (100–120 mCi) still warrant further research.

Tyrosine kinase inhibitors (TKI) has become the final treatment option for metastatic TC that is refractory to surgery or RAI, to improve the prognosis of patients with distant metastasis and progressive disease (59). According to previous literature, TKI is associated with cardiotoxicity (60). Cardiotoxicity may manifest as hypertension, heart failure, cardiac arrhythmias, thromboembolic events, fluid retention, or exacerbation of CAD. One RCT revealed a significantly increased risk of lenvatinib for CVD [RR 3.54 (95% CI: 1.03‒12.13)] (13). Nonetheless, the association between TKI and thyroid cancer still warrant further research.

Our meta-regression analysis demonstrated no statistically significant association between risk for CAD, cerebrovascular disease, Af and CVD mortality and mean age, women proportion, and follow up years. Whether age and sex effect the development of CVD in patients with TC remains unclear. A cohort study from UPDB revealed that TC survivors diagnosed at <40 years have an increased risk for several circulatory conditions when compared with the matched cancer-free population (27). Both patients with age < 40 or age ≥ 40 had increased risk for heart disease 1 to 10 years after cancer diagnosis (27). Only hypertension remained significant across 1 to >10 years after cancer diagnosis in both age groups, while diseases of the circulatory system remained significantly increased across 1 to >10 years for patients with age ≥ 40 (27). The association between TC and CVD in younger patients may be due to more RAI treatment (61–64) and TSH suppression therapy (65). TC survivors have been reported to have increased rates of distress and worry, especially in younger survivors (66, 67), which may contribute to the development of hypertension. A retrospective analysis from the SEER database indicated that male patients had poorer overall and cancer-specific survival (16). Male patients with TC had a larger tumor size and a larger proportion of metastasis, while female patients had a higher incidence and earlier age at diagnosis with TC (48.0 vs. 52.5 years old) (16). The better prognosis of TC in women is limited to the time of reproductive activity (68–70). Taken together, early detection of cardiovascular adverse effects in patients with TC appears to be justified in attempts to prevent CVD-related complications and mortality. The effect on CVD among different RAI dosage, levothyroxine dosage, TSH level, cancer stage and histology type still call for further research.

Our study has two advantages over prior meta-analyses. First, we included more qualified cohort studies. We also included RCTs due to lack of cohort studies. Second, the present study is the first meta-analysis to investigate the relationship between TC and RAI or lenvatinib. However, the evidence is still scarce. On the other hand, the present study had several limitations, the first of which was the limited number of eligible studies. Despite efforts to search using specific keywords to retrieve all relevant articles, the number of included studies was small. Further studies investigating the association between TC and CVD are needed to further optimize risk identification. Second, the heterogeneity of exposure measurement, end point ascertainment, statistical analysis (including cox proportional hazards model, standardized incidence ratio, and standardized mortality ratio), and data acquisition methods reduced our ability to compare the studies with one another. Pooled estimates could not be calculated on part of studies due to insufficient data. Future investigations are still needed to explore the potential causal pathways between exposure and outcome. Third, some of the studies lacked information of cancer parameters, such as cytology, staging, or TSH concentrations. Further understanding of the effects of treatment for TC on the cardiovascular system can be improved by stratifying detailed cancer information into covariates in future research. Fourth, despite the subgroups of each study were not overlapped, each of the subgroup was compared with the same control group to evaluate the risk for CVD. Finally, the meta-analysis could not exclude unmeasured residual confounders because most of the included evidence was derived from observational studies. However, the odds in these studies were adjusted for multiple relevant confounders to eliminate potential bias.

## Conclusion

TC was significantly associated with a higher risk for cerebrovascular disease and Af, although the hazard risk was not different between patients who did and did not undergo RAI ablation. Lenvatinib may increase CVD risk according to limited evidence. These results provide insights toward optimizing TC strategies that should consider the potential harms and benefits on cardiovascular health during cancer treatment.

## Data Availability

The original contributions presented in the study are included in the article/**Supplementary Material**, further inquiries can be directed to the corresponding author.
