# Supplementary material for: Association between thyroid cancer and cardiovascular disease: A meta-analysis
Source: Front Cardiovasc Med. 2023 Mar 3;10:1075844. doi: 10.3389/fcvm.2023.1075844 (PMC10020713; doi:10.3389/fcvm.2023.1075844)
Supplement: Supplementary file 1 [file Datasheet1.doc]

**Supplemental Table 1 Search term**

Pubmed

| (((((((((((((((((((((((((((((((((((((((((((((((((((((((Neoplasm, Thyroid) OR (Thyroid Neoplasm)) OR (Neoplasms, Thyroid)) OR (Thyroid Carcinoma)) OR (Carcinoma, Thyroid)) OR (Carcinomas, Thyroid)) OR (Thyroid Carcinomas)) OR (Cancer of Thyroid)) OR (Thyroid Cancers)) OR (Thyroid Cancer)) OR (Cancer, Thyroid)) OR (Cancers, Thyroid)) OR (Cancer of the Thyroid)) OR (Thyroid Cancer, Papillary)) OR (Cancer, Papillary Thyroid)) OR (Cancers, Papillary Thyroid)) OR (Papillary Thyroid Cancer)) OR (Papillary Thyroid Cancers)) OR (Thyroid Cancers, Papillary)) OR (Thyroid Carcinoma, Papillary)) OR (Carcinoma, Papillary Thyroid)) OR (Carcinomas, Papillary Thyroid)) OR (Papillary Thyroid Carcinomas)) OR (Thyroid Carcinomas, Papillary)) OR (Papillary Carcinoma Of Thyroid)) OR (Papillary Thyroid Carcinoma)) OR (Familial Nonmedullary Thyroid Cancer)) OR (Nonmedullary Thyroid Carcinoma)) OR (Carcinoma, Nonmedullary Thyroid)) OR (Carcinomas, Nonmedullary Thyroid)) OR (Nonmedullary Thyroid Carcinomas)) OR (Thyroid Carcinoma, Nonmedullary)) OR (Thyroid Carcinomas, Nonmedullary)) OR (Thyroid cancer, Hurthle cell)) OR (Hurthle Cell Thyroid Neoplasia)) OR (Follicular thyroid cancer, Hurthle cell type)) OR (Hurthle cell carcinoma of the thyroid)) OR (Thyroid carcinoma, Hurthle cell)) OR (Thyroid cancer, follicular, Hurthle cell type)) OR (Thyroid Carcinoma, Anaplastic)) OR (Anaplastic Thyroid Carcinoma)) OR (Anaplastic Thyroid Carcinomas)) OR (Carcinoma, Anaplastic Thyroid)) OR (Carcinomas, Anaplastic Thyroid)) OR (Thyroid Carcinomas, Anaplastic)) OR (Thyroid Cancer, Anaplastic)) OR (Anaplastic Thyroid Cancer)) OR (Anaplastic Thyroid Cancers)) OR (Cancer, Anaplastic Thyroid)) OR (Cancers, Anaplastic Thyroid)) OR (Thyroid Cancers, Anaplastic))) OR (Thyroid cancer, follicular)) OR (Adenocarcinoma, Follicular) |
| --- |
| (Strokes)) OR (Cerebrovascular Accident)) OR (Cerebrovascular Accidents)) OR (CVA (Cerebrovascular Accident))) OR (CVAs (Cerebrovascular Accident))) OR (Cerebrovascular Apoplexy)) OR (Apoplexy, Cerebrovascular)) OR (Vascular Accident, Brain)) OR (Brain Vascular Accident)) OR (Brain Vascular Accidents)) OR (Vascular Accidents, Brain)) OR (Cerebrovascular Stroke)) OR (Cerebrovascular Strokes)) OR (Stroke, Cerebrovascular)) OR (Strokes, Cerebrovascular)) OR (Apoplexy)) OR (Cerebral Stroke)) OR (Cerebral Strokes)) OR (Stroke, Cerebral)) OR (Strokes, Cerebral)) OR (Stroke, Acute)) OR (Acute Stroke)) OR (Acute Strokes)) OR (Strokes, Acute)) OR (Cerebrovascular Accident, Acute)) OR (Cerebrovascular Accident, Acute)) OR (Acute Cerebrovascular Accidents)) OR (Cerebrovascular Accidents, Acute)) OR (Atrial Fibrillation)) OR (Atrial Fibrillations)) OR (Fibrillation, Atrial)) OR (Fibrillations, Atrial)) OR (Auricular Fibrillation)) OR (Auricular Fibrillations)) OR (Fibrillation, Auricular)) OR (Fibrillations, Auricular)) OR (Persistent Atrial Fibrillation)) OR (Atrial Fibrillation, Persistent)) OR (Atrial Fibrillations, Persistent)) OR (Fibrillation, Persistent Atrial)) OR (Fibrillations, Persistent Atrial)) OR (Persistent Atrial Fibrillations)) OR (Familial Atrial Fibrillation)) OR (Atrial Fibrillation, Familial)) OR (Atrial Fibrillations, Familial)) OR (Familial Atrial Fibrillations)) OR (Fibrillation, Familial Atrial)) ) OR (Fibrillations, Familial Atrial)) OR (Paroxysmal Atrial Fibrillation)) OR (Atrial Fibrillation, Paroxysmal)) OR (Atrial Fibrillations, Paroxysmal)) OR (Fibrillation, Paroxysmal Atrial)) OR (Fibrillations, Paroxysmal Atrial)) OR (Paroxysmal Atrial Fibrillations)) OR (Heart Failure)) OR (Cardiac Failure)) OR (Heart Decompensation)) OR (Decompensation, Heart)) OR (Heart Failure, Right-Sided)) OR (Heart Failure, Right Sided)) OR (Right-Sided Heart Failure)) OR (Right Sided Heart Failure)) OR (Myocardial Failure)) OR (Congestive Heart Failure)) OR (Heart Failure, Congestive)) OR (Heart Failure, Left-Sided)) OR (Heart Failure, Left Sided)) OR (Left-Sided Heart Failure)) OR (Left Sided Heart Failure)) OR (Heart Failure, Diastolic)) OR (Diastolic Heart Failures)) OR (Heart Failures, Diastolic)) OR (Diastolic Heart Failure)) OR (Heart Failure, Systolic)) OR (Heart Failures, Systolic)) OR (Systolic Heart Failures)) OR (Systolic Heart Failure)) OR (Atherosclerosis)) OR (Atheroscleroses)) OR (Atherogenesis)) OR (Coronary Artery Disease)) OR (Artery Disease, Coronary)) OR (Artery Diseases, Coronary)) OR (Coronary Artery Diseases)) OR (Disease, Coronary Artery)) OR (Diseases, Coronary Artery) OR (Coronary Arteriosclerosis)) OR (Arterioscleroses, Coronary)) OR (Coronary Arterioscleroses)) OR (Atherosclerosis, Coronary)) OR (Atheroscleroses, Coronary)) OR (Coronary Atheroscleroses)) OR (Coronary Atherosclerosis)) OR (Arteriosclerosis, Coronary)) OR (Artery Disease, Carotid)) OR (Artery Diseases, Carotid)) OR (Carotid Artery Disease)) OR (Carotid Artery Disorders)) OR (Artery Disorder, Carotid)) OR (Artery Disorders, Carotid)) OR (Carotid Artery Disorder)) OR (Disorders, Carotid Artery)) OR (Arterial Diseases, Carotid)) OR (Arterial Disease, Carotid)) OR (Carotid Arterial Disease)) OR (Carotid Arterial Diseases)) OR (Carotid Atherosclerosis)) OR (Carotid Atheroscleroses)) OR (Carotid Atherosclerotic Disease)) OR (Atherosclerotic Diseases, Carotid)) OR (Carotid Atherosclerotic Diseases)) OR (Atherosclerotic Disease, Carotid)) OR (Internal Carotid Artery Diseases)) OR (Arterial Diseases, Internal Carotid)) OR (Arterial Diseases, Common Carotid)) OR (Common Carotid Artery Diseases)) OR (External Carotid Artery Diseases)) OR (Arterial Diseases, External Carotid)) OR (Intracranial Arteriosclerosis)) OR (Arterioscleroses, Intracranial)) OR (Arteriosclerosis, Intracranial)) OR (Intracranial Arterioscleroses)) OR (Intracranial Atherosclerosis)) OR (Atheroscleroses, Intracranial)) OR (Atherosclerosis, Intracranial)) OR (Intracranial Atheroscleroses)) OR (Cerebral Arteriosclerosis)) OR (Arterioscleroses, Cerebral)) OR (Arteriosclerosis, Cerebral)) OR (Cerebral Arterioscleroses)) OR (Cerebral Atherosclerosis)) OR (Atheroscleroses, Cerebral)) OR (Atherosclerosis, Cerebral)) OR (Cerebral Atheroscleroses)) OR (Hypertension, Pulmonary)) OR (Pulmonary Hypertension)) OR (Pulmonary Arterial Hypertension)) OR (Arterial Hypertension, Pulmonary)) OR (Hypertension, Pulmonary Arterial)) OR (Mitral Valve Prolapse)) OR (Mitral Valve Prolapses)) OR (Prolapse, Mitral Valve)) OR (Prolapses, Mitral Valve)) OR (Valve Prolapse, Mitral)) OR (Valve Prolapses, Mitral)) OR (Floppy Mitral Valve)) OR (Floppy Mitral Valves)) OR (Mitral Valve, Floppy)) OR (Mitral Valves, Floppy)) OR (Mitral Click-Murmur Syndrome)) OR (Click-Murmur Syndrome, Mitral)) OR (Mitral Click Murmur Syndrome)) OR (Syndrome, Mitral Click-Murmur)) OR (Systolic Click-Murmur Syndrome)) OR (Click-Murmur Syndrome, Systolic)) OR (Syndrome, Systolic Click-Murmur)) OR (Systolic Click Murmur Syndrome)) OR (Prolapsed Mitral Valve)) OR (Mitral Valve, Prolapsed)) OR (Mitral Valves, Prolapsed)) OR (Prolapsed Mitral Valves)) OR (Valve, Prolapsed Mitral)) OR (Valves, Prolapsed Mitral)) OR (Click-Murmur Syndrome)) OR (Click Murmur Syndrome)) OR (Click-Murmur Syndromes)) OR (Syndrome, Click-Murmur)) OR (Mitral Valve Prolapse Syndrome)) OR (Syndromes, Click-Murmur)) OR (Heart Valve Diseases)) OR (Disease, Heart Valve)) OR (Diseases, Heart Valve)) OR (Heart Valve Disease)) OR (Valve Disease, Heart)) OR (Valve Diseases, Heart)) OR (Valvular Heart Diseases)) OR (Disease, Valvular Heart)) OR (Diseases, Valvular Heart)) OR (Heart Disease, Valvular)) OR (Heart Diseases, Valvular)) OR (Valvular Heart Disease)) OR (Mitral Valve Insufficiency)) OR (Insufficiency, Mitral Valve)) OR (Valve Insufficiency, Mitral)) OR (Mitral Valve Regurgitation)) OR (Regurgitation, Mitral Valve)) OR (Valve Regurgitation, Mitral)) OR (Mitral Regurgitation)) OR (Regurgitation, Mitral)) OR (Mitral Valve Incompetence)) OR (Incompetence, Mitral Valve)) OR (Valve Incompetence, Mitral)) OR (Mitral Incompetence)) OR (Incompetence, Mitral)) OR (Mitral Insufficiency)) OR (Insufficiency, Mitral)) OR (Tricuspid Valve Insufficiency)) OR (Insufficiency, Tricuspid Valve)) OR (Valve Insufficiency, Tricuspid)) OR (Tricuspid Valve Regurgitation)) OR (Regurgitation, Tricuspid Valve)) OR (Valve Regurgitation, Tricuspid)) OR (Tricuspid Valve Incompetence)) OR (Incompetence, Tricuspid Valve)) OR (Valve Incompetence, Tricuspid)) OR (Tricuspid Incompetence)) OR (Incompetence, Tricuspid)) OR (Tricuspid Regurgitation)) OR (Regurgitation, Tricuspid)) OR (Pulmonary Embolism)) OR (Pulmonary Embolisms)) OR (Embolism, Pulmonary)) OR (Embolisms, Pulmonary)) OR (Pulmonary Thromboembolisms)) OR (Pulmonary Thromboembolism)) OR (Thromboembolism, Pulmonary)) OR (Thromboembolisms, Pulmonary)) OR (Venous Thrombosis)) OR (Phlebothrombosis)) OR (Phlebothromboses)) OR (Thrombosis, Venous)) OR (Thromboses, Venous)) OR (Venous Thromboses)) OR (Deep Vein Thrombosis)) OR (Deep Vein Thromboses)) OR (Thromboses, Deep Vein)) OR (Vein Thromboses, Deep)) OR (Vein Thrombosis, Deep)) OR (Deep-Venous Thrombosis)) OR (Deep-Venous Thromboses)) OR (Thromboses, Deep-Venous)) OR (Thrombosis, Deep-Venous)) OR (Deep-Vein Thrombosis)) OR (Deep-Vein Thromboses)) OR (Thromboses, Deep-Vein)) OR (Thrombosis, Deep-Vein)) OR (Thrombosis, Deep Vein)) OR (Deep Venous Thrombosis)) OR (Deep Venous Thromboses)) OR (Thromboses, Deep Venous)) OR (Thrombosis, Deep Venous)) OR (Venous Thromboses, Deep)) OR (Venous Thrombosis, Deep) OR (Mortalities) OR (Case Fatality Rate)) OR (Case Fatality Rates)) OR (Rate, Case Fatality)) OR (Rates, Case Fatality)) OR (CFR Case Fatality Rate)) OR (Crude Death Rate)) OR (Crude Death Rates)) OR (Death Rate, Crude)) OR (Rate, Crude Death)) OR (Crude Mortality Rate)) OR (Crude Mortality Rates)) OR (Mortality Rate, Crude)) OR (Rate, Crude Mortality)) OR (Death Rate)) OR (Death Rates)) OR (Rate, Death)) OR (Mortality Rate)) OR (Mortality Rates)) OR (Rate, Mortality)) OR (Mortality, Excess)) OR (Excess Mortality)) OR (Excess Mortalities)) OR (Decline, Mortality)) OR (Mortality Declines)) OR (Mortality Decline)) OR (Mortality Determinants)) OR (Determinants, Mortality)) OR (Determinant, Mortality)) OR (Mortality Determinant)) OR (Mortality, Differential)) OR (Differential Mortality)) OR (Differential Mortalities)) OR (Age-Specific Death Rate)) OR (Age-Specific Death Rates)) OR (Death Rate, Age-Specific)) OR (Rate, Age-Specific Death)) OR (Age Specific Death Rate)) |

Embase

| cancer, AND thyroid AND gland OR (thyroid AND gland AND cancer) OR (thyroid AND malignant AND tumor) OR (thyroid AND malignant AND tumour) OR (thyroidal AND cancer) OR (thyroidal AND gland AND cancer) OR (thyroid AND carcinoma) |
| --- |
| 'atrium fibrillation'/exp OR 'atrium fibrillation' OR (('atrium'/exp OR atrium) AND ('fibrillation'/exp OR fibrillation)) OR (auricular AND fibrilation) OR (auricular AND fibrillation) OR (cardiac AND atrial AND fibrillation) OR (cardiac AND atrium AND fibrillation) OR (fibrillation, AND heart AND atrium) OR (heart AND atrial AND fibrillation) OR (heart AND atrium AND fibrillation) OR (heart AND fibrillation AND atrium) OR ('non valvular' AND atrial AND fibrillation) OR (nonvalvular AND atrial AND fibrillation) OR (backward AND failure, AND heart) OR (cardiac AND backward AND failure) OR (cardiac AND decompensation) OR (cardiac AND failure) OR (cardiac AND incompetence) OR (cardiac AND insufficiency) OR (cardiac AND stand AND still) OR (cardial AND decompensation) OR (cardial AND insufficiency) OR (chronic AND heart AND failure) OR (chronic AND heart AND insufficiency) OR (decompensatio AND cordis) OR (decompensation, AND heart) OR (heart AND backward AND failure) OR (heart AND decompensation) OR (heart AND incompetence) OR (heart AND insufficiency) OR (insufficientia AND cardis) OR (myocardial AND failure) OR (myocardial AND insufficiency) OR (essential AND pulmonary AND hypertension) OR (hypertension, AND lung) OR (hypertension, AND pulmonary) OR (hypertensive AND pulmonary AND vascular AND disease) OR (lung AND arterial AND hypertension) OR (lung AND artery AND hypertension) OR (lung AND hypertension) OR (primary AND pulmonary AND hypertension) OR (pulmonary AND arterial AND hypertension) OR (pulmonary AND artery AND hypertension) OR (pulmonary AND fixed AND hypertension) OR (pulmonary AND hypertensive AND disease) OR (pulmonary AND hypertensive AND diseases) OR (pulmonary AND hypertensive AND disorder) OR (pulmonary AND hypertensive AND disorders) OR (mitral AND prolapse) OR (mitral AND valvular AND prolapse) OR (billowing AND mitral AND leaflet AND syndrome) OR (bicuspid AND cardiac AND valve AND incompetence) OR (bicuspid AND cardiac AND valve AND insufficiency) OR (bicuspid AND cardiac AND valve AND regurgitation) OR (bicuspid AND heart AND valve AND incompetence) OR (bicuspid AND heart AND valve AND insufficiency) OR (bicuspid AND heart AND valve AND regurgitation) OR (bicuspid AND incompetence) OR (bicuspid AND insufficiency) OR (bicuspid AND regurgitation) OR (bicuspid AND valve AND insufficiency) OR (bicuspid AND valve AND regurgitation) OR (bicuspid AND valvular AND incompetence) OR (bicuspid AND valvular AND insufficiency) OR (bicuspid AND valvular AND regurgitation) OR (heart AND valve AND incompetence, AND mitral) OR (heart AND valve AND insufficiency, AND mitral) OR (heart AND valve AND regurgitation, AND mitral) OR (incompetence, AND mitral AND valve) OR (left AND atrioventricular AND cardiac AND valve AND incompetence) OR (left AND atrioventricular AND cardiac AND valve AND insufficiency) OR (left AND atrioventricular AND cardiac AND valve AND regurgitation) OR (left AND atrioventricular AND cardiac AND valvular AND incompetence) OR (left AND atrioventricular AND heart AND valve AND incompetence) OR (left AND atrioventricular AND heart AND valve AND insufficiency) OR (left AND atrioventricular AND heart AND valve AND regurgitation) OR (left AND atrioventricular AND incompetence) OR (left AND atrioventricular AND insufficiency) OR (left AND atrioventricular AND regurgitation) OR (left AND atrioventricular AND valve AND incompetence) OR (left AND atrioventricular AND valve AND insufficiency) OR (left AND atrioventricular AND valve AND regurgitation) OR (mitral AND cardiac AND valve AND incompetence) OR (mitral AND cardiac AND valve AND insufficiency) OR (mitral AND cardiac AND valve AND regurgitation) OR (mitral AND heart AND valve AND incompetence) OR (mitral AND heart AND valve AND insufficiency) OR (mitral AND heart AND valve AND regurgitation) OR (mitral AND incompetence) OR (mitral AND insufficiency) OR (mitral AND paravalvular AND regurgitation) OR (mitral AND regurgitation) OR (mitral AND valve AND incompetence) OR (mitral AND valve AND insufficiency) OR (mitral AND valvular AND incompetence) OR (mitral AND valvular AND insufficiency) OR (mitral AND valvular AND regurgitation) OR (mitralis AND regurgitation) OR (regurgitation, AND mitral AND valve) OR (valve AND incompetence, AND mitral) OR (valve AND regurgitation, AND mitral) OR atherosclerosis OR (coronary AND disease) OR (coronary AND artery AND disease) OR (brain AND vascular AND disease) OR (brain AND vasculopathy) OR (cerebral AND small AND vessel AND disease) OR (cerebral AND small AND vessel AND diseases) OR (cerebral AND vascular AND disease) OR (cerebral AND vascular AND disorder) OR (cerebral AND vascular AND disturbance) OR (cerebral AND vascular AND lesion) OR (cerebral AND vasculopathy) OR (cerebrovascular AND damage) OR (cerebrovascular AND disorder) OR (cerebrovascular AND disorders) OR (cerebrovascular AND lesion) OR (cerebrovascular AND pathology) OR (cerebrovascular AND syndrome) |

**Supplemental Table 2** PECOS framework

| PECOS framework | Eligibility criteria |
| --- | --- |
| Population | All patients with thyroid cancer |
| Exposure | Thyroidectomy, thyroxine, RAI or TKIs |
| Comparison/control | Healthy population |
| Outcome | Coronary artery disease, atrial fibrillation, cerebrovascular disease, cardiovascular disease mortality |

**Supplemental Table 3 Risk of bias assessment**

|  | Confounding | Selection of participants into the study | Measurement of the exposure | Deviations from intended exposures | Missing data | Measurement of outcomes | Selection of the reported result | Overall risk of bias |
| --- | --- | --- | --- | --- | --- | --- | --- | --- |
| Hesselink, 2013 (1) | Low | Low | Low | Low | Some concerns | Low | Low | Some concerns |
| Hesselink, 2015 (2) | Low | Low | Low | Low | Low | Low | Low | Low |
| Schlumberger, 2015 (3) | Low | Low | Low | Low | Low | Low | Low | Low |
| Lin, 2017 (4) | Low | Low | Low | Low | Some concerns | Low | Low | Some concerns |
| Blackburn, 2017 (5) | Low | Low | Low | Low | Low | Low | Low | Low |
| Pajamäki, 2018 (6) | Low | Low | Low | Low | Some concerns | Low | Low | Some concerns |
| Suh, 2019 (7) | Low | Low | Low | Low | Some concerns | Low | Low | Some concerns |
| Izkhakov, 2019 (8) | Low | Low | Low | Low | Low | Low | Low | Low |
| Toulis, 2019 (9) | Low | Low | Low | Low | Some concerns | Low | Low | Some concerns |
| Du, 2021 (10) | Low | Low | Low | Low | Low | Low | Low | Low |
| Kim, 2020 (11) | Low | Low | Low | Low | Some concerns | Low | Low | Some concerns |
| Zoltek, 2020 (12) | Low | Low | Low | Low | Low | Low | Low | Low |
| Lu, 2021 (13) | Low | Low | Low | Low | Some concerns | Low | Low | Some concerns |
| Kao, 2021 (14) | Low | Low | Low | Low | Low | Low | Low | Low |
| Leboulleux, 2022 (15) | Low | Low | Low | Low | Low | Low | Low | Low |

**Supplemental Figure 1 Contour-enhanced funnel plot of the publication bias**

**
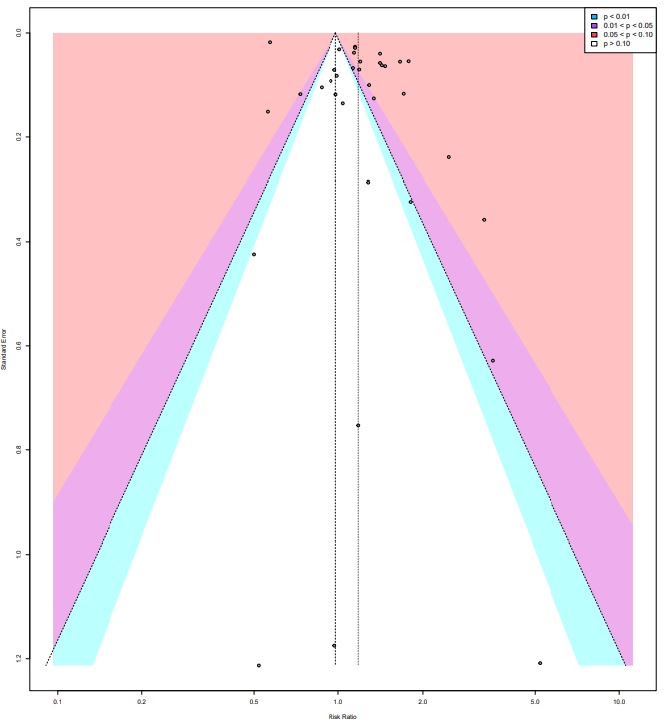
**

**Supplemental Figure 2 Bubble plot of meta-regression between the proportion of women and the risk of (A) coronary artery disease; (B) cerebrovascular disease; (C) atrial fibrillation; (D) CVD mortality among patients with thyroid cancer compared with general health population**

**
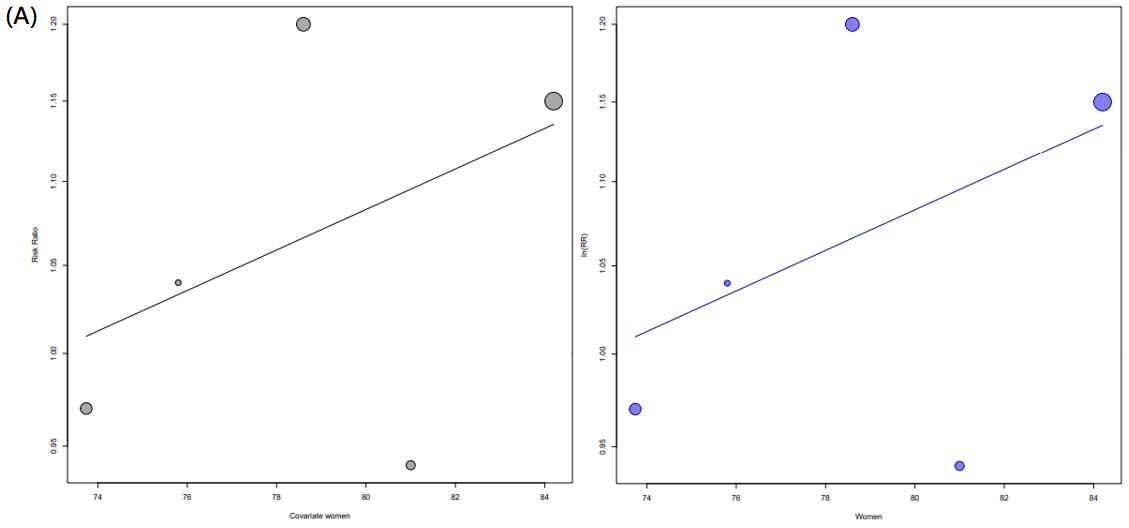
**

**
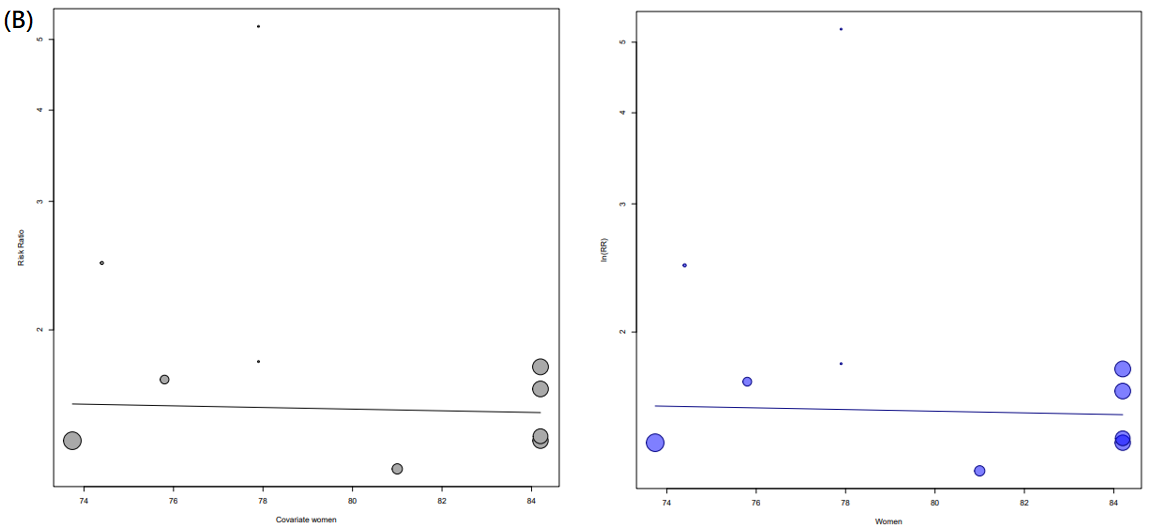
**

**
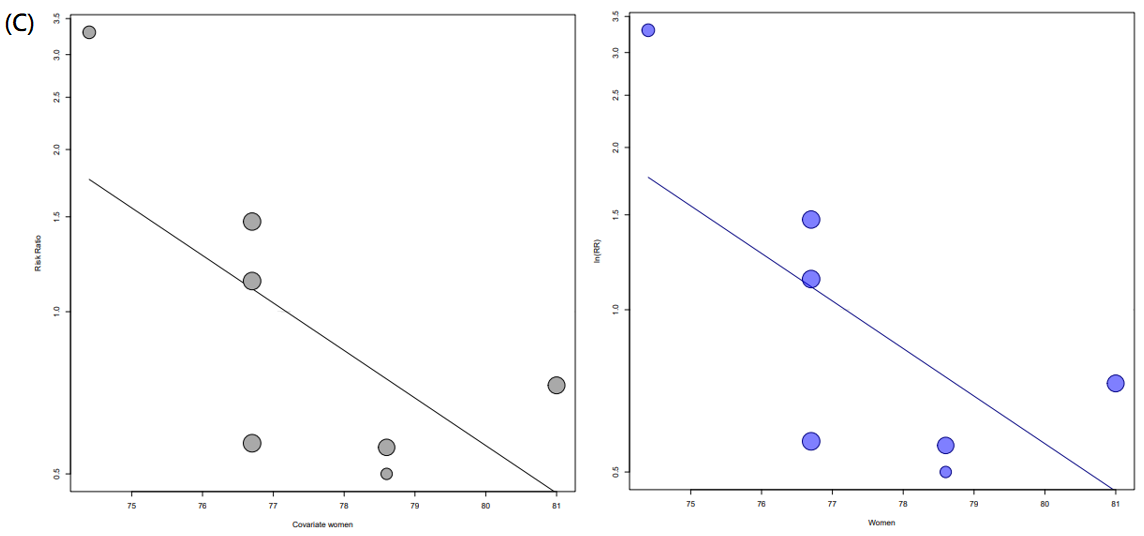
**

**
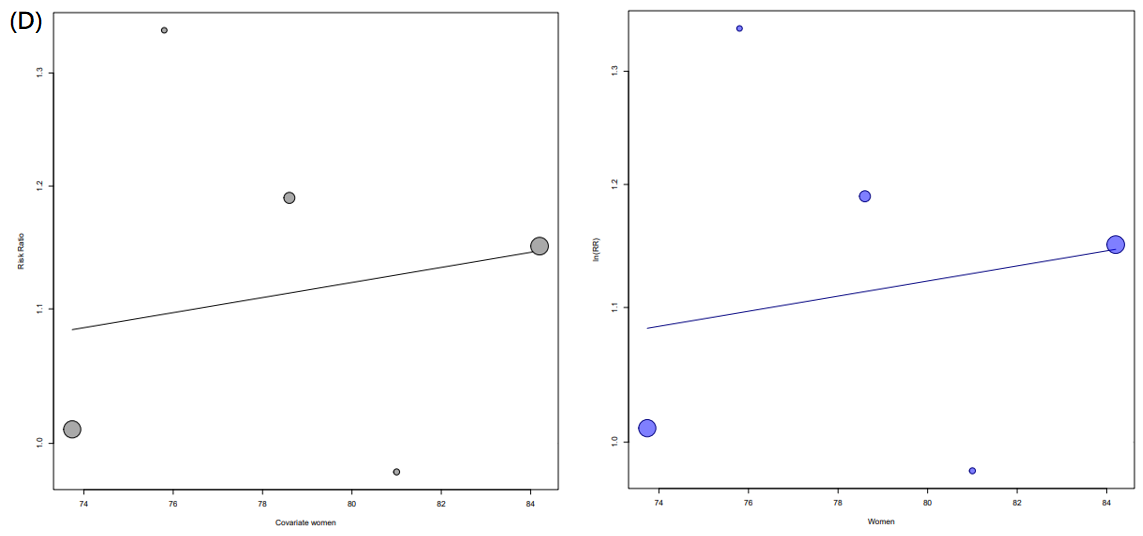
**

**Supplemental Figure 3 Bubble plot of meta-regression between mean age and the risk of (A) coronary artery disease; (B) cerebrovascular disease; (C) atrial fibrillation; (D) CVD mortality among patients with thyroid cancer compared with general health population**

**
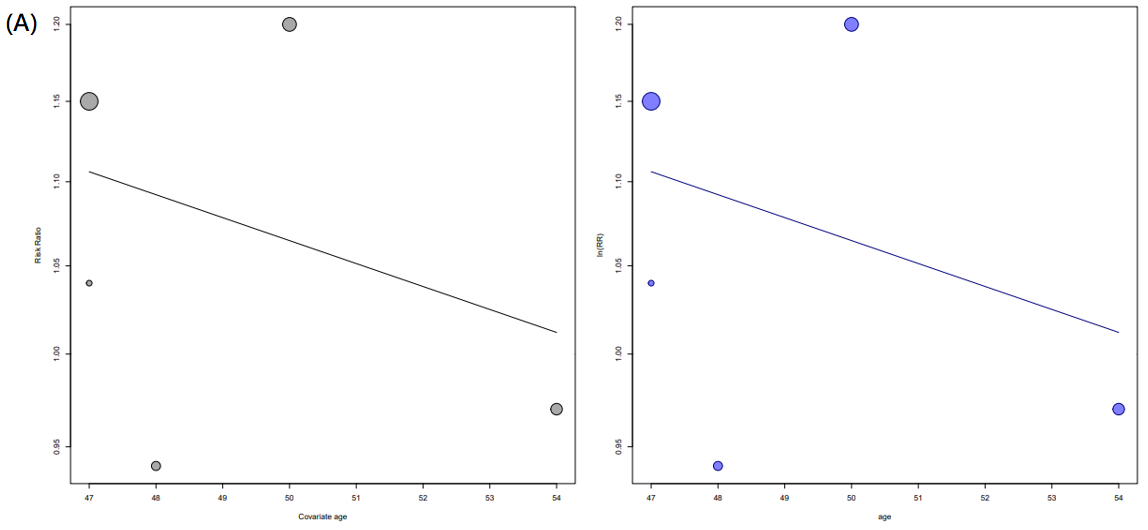
**

**
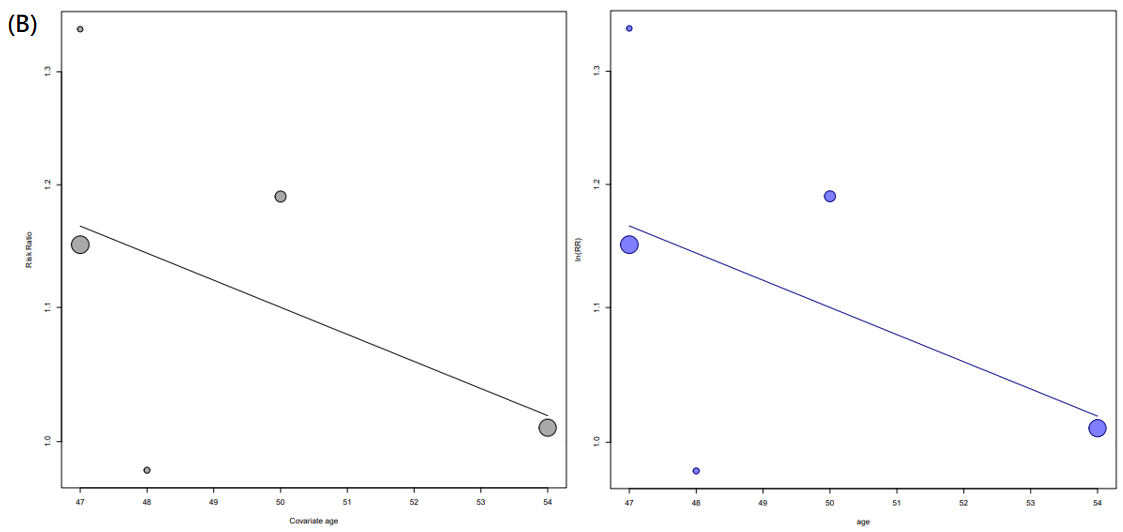
**

**
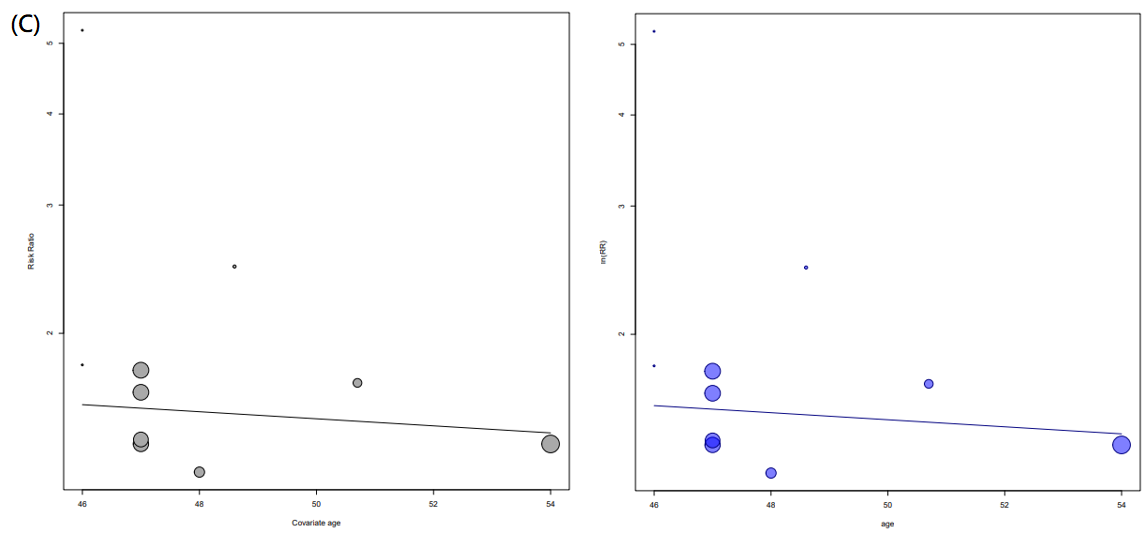
**

**
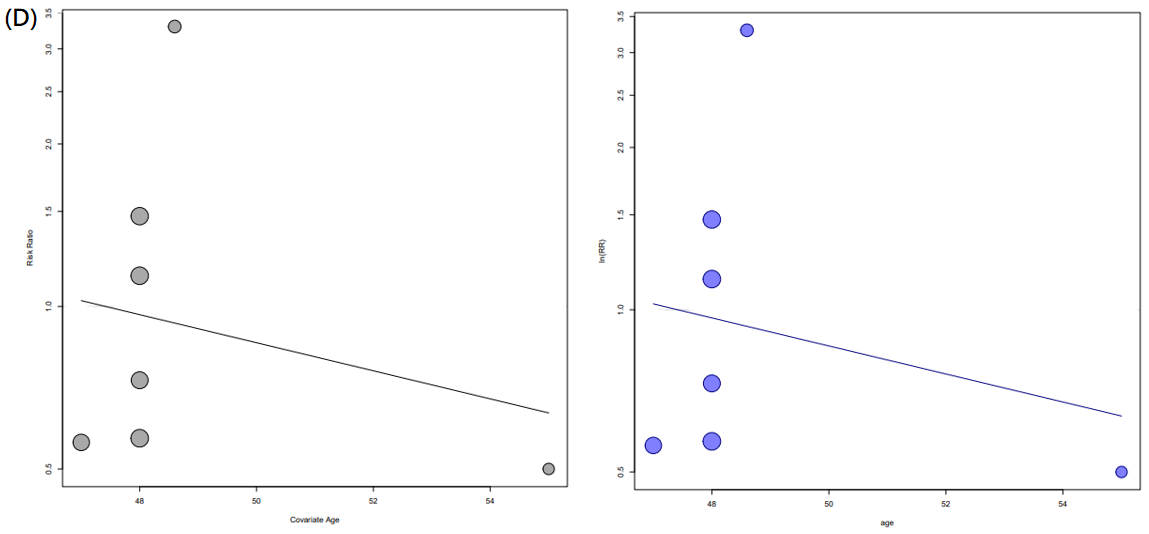
**

**References**

1. Klein Hesselink EN, Klein Hesselink MS, de Bock GH, Gansevoort RT, Bakker SJ, Vredeveld EJ, et al. Long-term cardiovascular mortality in patients with differentiated thyroid carcinoma: an observational study. *J Clin Oncol.* (2013) 31:4046-53. doi: 10.1200/JCO.2013.49.1043.

2. Klein Hesselink EN, Lefrandt JD, Schuurmans EP, Burgerhof JG, Groen B, Gansevoort RT, et al. Increased Risk of Atrial Fibrillation After Treatment for Differentiated Thyroid Carcinoma. *J Clin Endocrinol Metab*. (2015) 100:4563-9. doi: 10.1210/jc.2015-2782.

3. Schlumberger M, Tahara M, Wirth LJ, Robinson B, Brose MS, Elisei R, et al. Lenvatinib versus placebo in radioiodine-refractory thyroid cancer. *N Engl J Med*. (2015) 372:621-30. doi: 10.1056/NEJMoa1406470.

4. Lin CY, Lin CL, Lo YC, Kao CH. Association between radioiodine treatment for thyroid cancer and risk of stroke. *Head* *Neck*. (2017) 39:2311-8. doi: 10.1002/hed.24903.

5. Blackburn BE, Ganz PA, Rowe K, Snyder J, Wan Y, Deshmukh V, et al. Aging-Related Disease Risks among Young Thyroid Cancer Survivors. *Cancer Epidemiol Biomarkers Prev.* (2017) 26:1695-704. doi: 10.1158/1055-9965.EPI-17-0623.

6. Pajamäki N, Metso S, Hakala T, Ebeling T, Huhtala H, Ryödi E, et al. Long-term cardiovascular morbidity and mortality in patients treated for differentiated thyroid cancer. *Clin Endocrinol (Oxf)*. (2018) 88:303-10. doi: 10.1111/cen.13519.

7. Suh B, Shin DW, Park Y, Lim H, Yun JM, Song SO, et al. Increased cardiovascular risk in thyroid cancer patients taking levothyroxine: a nationwide cohort study in Korea. *Eur J Endocrinol*. (2019) 180:11-20. doi: 10.1530/EJE-18-0551.

8. Izkhakov E, Meyerovitch J, Barchana M, Shacham Y, Stern N, Keinan-Boker L. Long-term cardiovascular and cerebrovascular morbidity in Israeli thyroid cancer survivors. *Endocr Connect*. (2019) 8:398-406. doi: 10.1530/EC-19-0038.

9. Toulis KA, Viola D, Gkoutos G, Keerthy D, Boelaert K, Nirantharakumar K. Risk of incident circulatory disease in patients treated for differentiated thyroid carcinoma with no history of cardiovascular disease. *Clin Endocrinol (Oxf)*. (2019) 91:323-30. doi: 10.1111/cen.13990.

10. Du B, Wang F, Wu L, Wang Z, Zhang D, Huang Z, et al. Cause-specific mortality after diagnosis of thyroid cancer: a large population-based study. *Endocrine*. (2021) 72:179-89. doi: 10.1007/s12020-020-02445-8.

11. Kim KJ, Song JE, Kim JY, Bae JH, Kim NH, Yoo HJ, et al. Effects of radioactive iodine treatment on cardiovascular disease in thyroid cancer patients: a nationwide cohort study. *Ann Transl Med*. (2020) 8:1235. doi: 10.21037/atm-20-5222.

12. Zoltek M, Andersson TM, Hedman C, Ihre-Lundgren C, Nordenvall C. Cardiovascular Incidence in 6900 Patients with Differentiated Thyroid Cancer: a Swedish Nationwide Study. *World J Surg*. (2020) 44:436-41. doi: 10.1007/s00268-019-05249-8.

13. Lu YL, Lin SF, Wu MH, Lee YY, Lee PW, Chang SH, et al. Survival and Death Causes in Thyroid Cancer in Taiwan: A Nationwide Case-Control Cohort Study. *Cancers*. (2021) 13:3955. doi: 10.3390/cancers13163955.

14. Kao CH, Chung CH, Chien WC, Shen DH, Lin LF, Chiu CH, et al. Radioactive Iodine Treatment and the Risk of Long-Term Cardiovascular Morbidity and Mortality in Thyroid Cancer Patients: A Nationwide Cohort Study. *J Clin Med*. (2021) 10:4032. doi: 10.3390/jcm10174032

15. Leboulleux S, Bournaud C, Chougnet CN, Zerdoud S, Al Ghuzlan A, Catargi B, et al. Thyroidectomy without Radioiodine in Patients with Low-Risk Thyroid Cancer. *N Engl J Med*. (2022) 386:923-32. doi: 10.1056/NEJMoa2111953
